# Visual DAT‐SPECT Outperforms Semiquantitative Analysis in a “Pseudo‐SWEDD” Case of Benign Tremulous Parkinsonism

**DOI:** 10.1002/mdc3.70374

**Published:** 2025-10-03

**Authors:** Daniele Birreci, Luca Angelini, Martina De Riggi, Simone Aloisio, Adriana Martini, Sofia A. Grandolfo, Luca Marsili, Roberto Cilia, Alberto J. Espay, Matteo Bologna

**Affiliations:** ^1^ Department of Human Neurosciences Sapienza University of Rome Rome Italy; ^2^ IRCCS Neuromed Pozzilli (IS) Italy; ^3^ James J. and Joan A. Gardner Family Center for Parkinson's Disease and Movement Disorders Department of Neurology, University of Cincinnati Cincinnati Ohio USA; ^4^ Parkinson and Movement Disorders Unit, Department of Clinical Neurosciences Fondazione IRCCS Istituto Neurologico Carlo Besta Milan Italy

**Keywords:** parkinsonism, levodopa, kinematic analysis, normal dopaminergic imaging

A subset of patients with a long‐standing history of hand tremor may eventually develop features of Parkinson's disease (PD). This monosymptomatic presentation, termed “benign tremulous parkinsonism” (BTP), may exhibit postural tremor reminiscent of essential tremor (ET) with a resting component.^1^ In such cases, dopamine transporter single‐photon emission computed tomography (DAT‐SPECT) may help distinguish ET from PD. We report a case in which clinical and neurophysiological findings supported PD, whereas quantitative DAT‐SPECT analysis did not.

A 73‐year‐old man, referred to the outpatient clinic at Sapienza University of Rome, had a 10‐year history of a slowly progressing right‐predominant bilateral action and rest hand tremor (Appendix S1). On examination, he showed a re‐emergent component of the hand tremor, head and voice tremor, hypomimia, subtle bradykinesia with rigidity on the right side associated with a sequence effect and slowed speed on the right‐arm finger‐to‐nose task (Video 1).

**Video 1:** The clinical evaluation of the patient treated with low‐dose levodopa (first segment) and untreated (second segment). Each segment includes the assessment of the patient with arms at rest, during the maintenance of 2 postures, the finger‐to‐nose task, the finger‐tapping task, the Archimedes spirals task, the pouring test, and a gait evaluation.

Despite a clinical impression of parkinsonism, the semiquantitative imaging assessment using DaTQUANT (Appendix S2) showed tracer uptake within the lower end of the normal range^2^ (Fig. 1), justifying a normal DAT‐SPECT report. Visual inspection, however, detected modest DAT signal reduction in the left posterior putamen, contralateral to the tremor side. At the time of assessment, the patient was taking levodopa (l‐dopa)/benserazide (100/25 mg), 1 tablet thrice daily. Although he reported no benefits, we conducted a clinical and kinematic evaluation before and after treatment discontinuation. The comparison between the *on* and *off* states is shown in Video 1, even if no significant differences in clinical scores were documented, likely due to a subtherapeutic dose (Appendix S1). Nevertheless, kinematic analysis^3^ showed a subclinical l‐dopa effect, with reduced re‐emergent tremor amplitude and improved movement velocity and sequence effect (Fig. 1; Table S1).

**FIG. 1:**
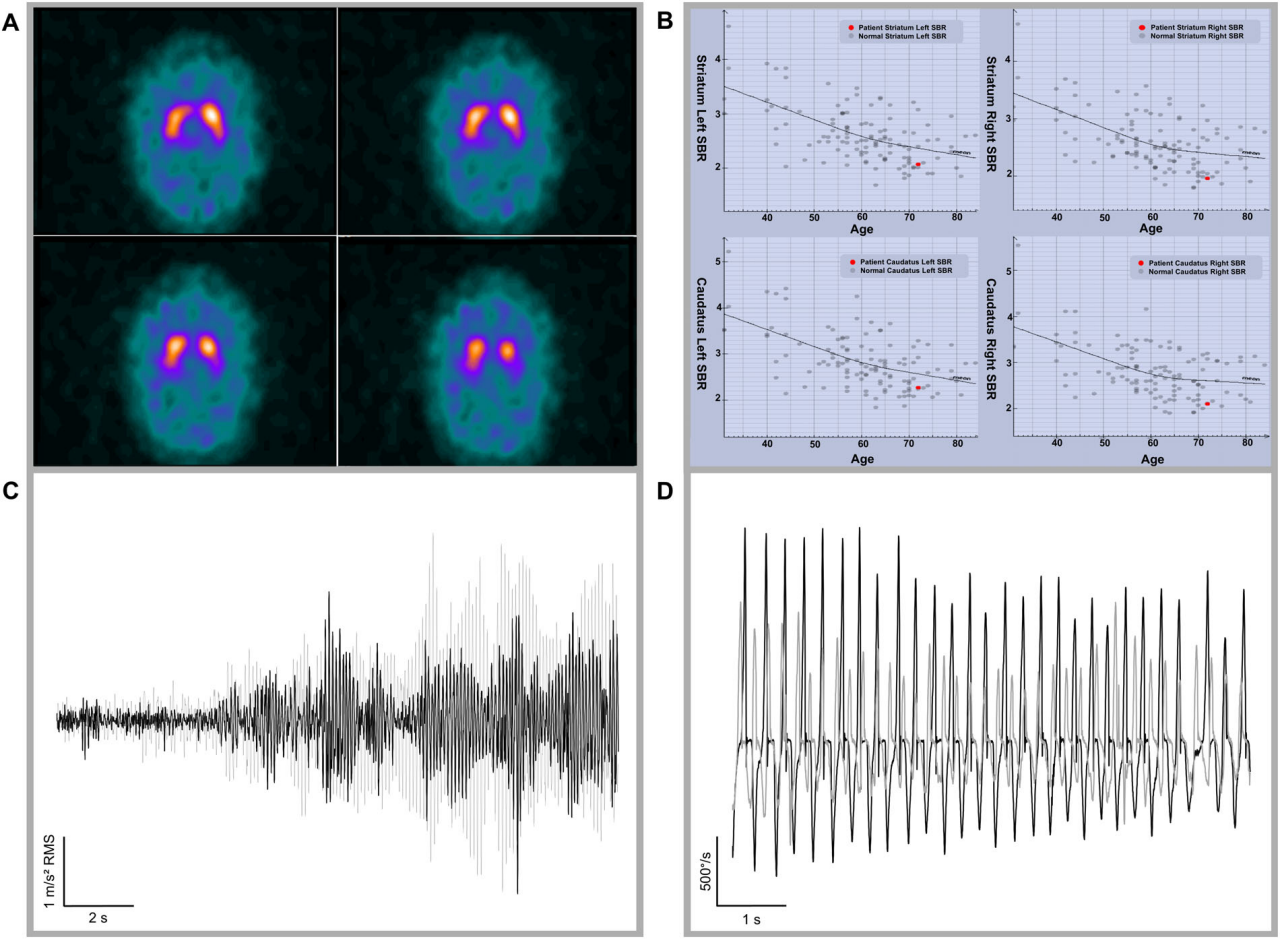
Dopamine transporter single‐photon emission computed tomography (DAT‐SPECT) data and kinematic traces of tremor and bradykinesia. (**A**) DAT‐SPECT images of the radioligand uptake, showing consistent reduction in the left posterior putamen. (**B**) Scatterplots of striatal binding ratio (SBR) values of the right and left striatum (top panels) and caudate (bottom panels) as a function of age. The solid black lines represent normative data across different age groups. Each gray dot represents an individual from the reference population, whereas the red dots correspond to the patient values: right striatum SBR = 1.96 (z‐score: −1.08), left striatum SBR = 2.07 (z‐score: −0.70); right caudate SBR = 2.10 (z‐score: −1.04), left caudate SBR = 2.27 (z‐score: −0.60). The analysis also yielded derived SBR values for the putamen: right = 1.90 (z‐score: −1.04), left = 1.96 (z‐score: −0.72), putamen‐to‐caudate uptake ratio bilaterally (right: 0.90, left: 0.86). (**C**) Kinematic traces of re‐emergent tremor on the right side (more affected), expressed as the root mean square (RMS) of the acceleration traces (m/s^2^); the black line represents the recording with levodopa (l‐dopa), whereas the light‐gray line represents the recording *off* l‐dopa. (**D**) Kinematic traces of finger‐tapping velocity on the right side (more affected), expressed in degrees per second; the black line represents the recording with l‐dopa, whereas the light‐gray line represents the recording *off* l‐dopa. Observe the progressive velocity reduction consistent with a sequence effect.

To the best of our knowledge, this is the first case where the clinical examination suggested PD, a kinematic analysis suggested a subclinical l‐dopa effect, a visual DAT‐SPECT interpretation supported such conclusion, but a quantitative analysis did not—in the end justifying the incorrect conclusion of a scan without evidence of dopaminergic denervation (SWEDD). This “pseudo‐SWEDD” case highlights the dissociation between qualitative and semiquantitative DAT‐SPECT appraisal and challenges the presumed superiority of the latter.

Collectively, the data point to a BTP presentation of PD. Prior studies have reported subtle bilateral DAT reduction in tremor‐dominant PD^4^ and relatively preserved caudate binding versus non‐tremor PD,^5^ suggesting milder dopaminergic deficits in such phenotypes, possibly shared by BTP. Common misdiagnoses include ET, dystonic tremor, or “SWEDD.”

Particularly, the kinematic analysis demonstrated a subclinical improvement in motor function with subtherapeutic doses of l‐dopa (a “sub‐*on*” state), whereas its discontinuation worsened the re‐emergent tremor and slowed movement execution without the patient's awareness, supporting the diagnosis of PD.

In conclusion, this case illustrates how integrated clinical and kinematic assessments, along with qualitative DAT‐SPECT inspection, can support the diagnosis of PD despite normal quantitative imaging reports.

## Author Roles

(1) Research project: A. Conception, B. Organization, C. Execution; (2) Statistical analysis: A. Design, B. Execution, C. Review and critique; (3) Manuscript: A. Writing of the first draft, B. Review and critique.

D.B.: 1A, 1B, 1C, 3A

L.A.: 1A, 3B

M.D.R.: 3B

S.A.: 3B

A.M.: 1C, 3B

S.A.G.: 3B

L.M.: 1A, 3B

R.C.: 1A, 3B

A.J.E.: 1A, 3A, 3B

M.B.: 1A, 1B, 3A, 3B

## Disclosures

**Ethical Compliance Statement:** The authors confirm that approval of an institutional review board or ethics committee was not required for this case report. All procedures performed were in accordance with the ethical standards of our institution and with the Helsinki Declaration. Written informed consent was obtained from the patient for the publication of his data and the online distribution of the related video material. We confirm that we have read the journal's position on issues involved in ethical publication and affirm that this work is consistent with those guidelines.

**Funding Sources and Conflicts of Interest:** This work was supported by the Italian Ministry of Health (Current Research 2025). The authors declare that there are no conflicts of interest relevant to this work.

**Financial Disclosures for the Previous 12 Months:** L.M. has received honoraria from the International Association of Parkinsonism and Related Disorders (IAPRD) Society for social media and web support. L.M.has received a grant (collaborative research agreement) from the International Parkinson and Movement Disorders Society for the MDS‐UTRS Validation Program (role: principal investigator), Non‐Profit. A.E.J. has received grant support from the NIH and the Michael J. Fox Foundation; personal compensation as a consultant/scientific advisory board member for Mitsubishi Tanabe Pharma America (formerly Neuroderm), Amneal, Acorda, AbbVie, Bial, Kyowa Kirin, Supernus (formerly USWorldMeds), NeuroDiagnostics, Inc. (SYNAPS Dx), Intrance Medical Systems, Inc., Merz, Praxis Precision Medicines, Citrus Health, and Herantis Pharma; Data Safety Monitoring Board (chair) of AskBio; and publishing royalties from Lippincott Williams & Wilkins, Cambridge University Press, and Springer. He is coinventor of the patent “Compositions and methods for treatment and/or prophylaxis of proteinopathies.” He cofounded REGAIN Therapeutics to fund preclinical studies but relinquished the right to any personal income from future treatments. The other authors declare that there are no additional disclosures to report.

## Supporting information


**Table S1.** Kinematic measures during levodopa therapy (*on* state) and after treatment discontinuation (*off* state), including tremor amplitude and frequency, and finger‐tapping parameters, with reference values from healthy controls.
**Appendix S1.** Additional clinical details, including past medical history and results of clinical assessments during levodopa therapy (*on* state) and after treatment discontinuation (*off* state), with clinical scores.
**Appendix S2.** Description of the dopamine transporter single‐photon emission computed tomography (DAT‐SPECT) semiquantitative analysis using DaTQUANT, including software specifications, image processing methods, regions of interest analyzed, reference region used for normalization, and the normative database applied for z‐score calculation.

## Data Availability

The data that support the findings of this study are available on request from the corresponding author. The data are not publicly available due to privacy or ethical restrictions.
