# Supplementary material for: Visual DAT‐SPECT Outperforms Semiquantitative Analysis in a “Pseudo‐SWEDD” Case of Benign Tremulous Parkinsonism
Source: Mov Disord Clin Pract. 2025 Oct 3;13(3):824–6. doi: 10.1002/mdc3.70374 (PMC13042594; doi:10.1002/mdc3.70374)
Supplement: Supplementary file 2 — Table S1. Kinematic measures during levodopa therapy (on state) and after treatment discontinuation (off state), including tremor amplitude and frequency, and finger‐tapping parameters, with reference values from healthy controls. Appendix S1. Additional clinical details, including past medical history and results of clinical assessments during levodopa therapy (on state) and after treatment discontinuation (off state), with clinical scores. Appendix S2. Description of the dopamine transporter single‐photon emission computed tomography (DAT‐SPECT) semiquantitative analysis using DaTQUANT, including software specifications, image processing methods, regions of interest analyzed, reference region used for normalization, and the normative database applied for z‐score calculation. [file MDC3-13-824-s001.docx]

|  | **Right side**  **ON left** | | **Left side** | |
| --- | --- | --- | --- | --- |
|  | **on** | **off** | **on** | **off** |
| Tremor |  |  |  |  |
| Rest tremor |  |  |  |  |
| Amplitude | 0.53 | 0.54 | 0.04 | 0.03 |
| Frequency | 4.50 | 4.52 | 4.62 | 4.59 |
| Re-emergent tremor |  |  |  |  |
| Amplitude | 0.15 | 0.29 | 0.04 | 0.08 |
| Frequency | 4.89 | 5.22 | 5.06 | 5.03 |
| Kinetic tremor |  |  |  |  |
| CI | 1.09 | 1.06 | 1.12 | 1.06 |
| D/A | 0.56 | 0.69 | 0.59 | 0.58 |
| Head tremor |  |  |  |  |
| Amplitude | 0.35 | 0.32 | 0.35 | 0.32 |
| Frequency | 6.03 | 6.06 | 6.03 | 6.06 |
| Finger-tapping |  |  |  |  |
| N° mov | 36.00 | 29.67 | 30.67 | 25.25 |
| Velocity | 1215.91 | 1012.17 | 1358.25 | 1152.77 |
| Amplitude | 52.72 | 52.62 | 61.60 | 62.69 |
| CV | 0.12 | 0.07 | 0.06 | 0.11 |
| Velocity slope | -23.32 | -19.68 | -21.67 | -20.40 |
| Amplitude slope | -0.11 | -0.29 | -0.24 | -0.57 |

**Supplementary table 1. Kinematic measures during levodopa therapy (*on* state) and after treatment discontinuation (*off* state).**

Amplitude for rest, re-emergent and head tremor is expressed in m/s² RMS, while frequency is reported in Hz. For finger-tapping, velocity is expressed in degrees/s, amplitude in degrees, velocity slope in (degrees/s)/number of movements, and amplitude slope in degrees/number of movements. The mean values ± standard deviations in healthy controls are 46.26 ± 16.41 for number of movements, 1080.80 ± 252.83 for velocity, 49.93 ± 13.48 for amplitude, 0.09 ± 0.03 for dysrhythmia, -6.39 ± 4.73 for velocity reduction and -0.13 ± 0.21 for amplitude reduction. Abbreviations: CI, curvature index; D/A, deceleration/acceleration ratio; N° mov, number of movements; CV, coefficient of variation.

**Supplementary appendix 1. Additional clinical details**

The patient’s past medical history included parathyroidectomy for adenoma, post-ischemic dilated cardiomyopathy treated with a Pacemaker–Implantable Cardioverter Defibrillator (PMK-ICD), chronic renal failure, dyslipidemia, and insulin-dependent diabetes mellitus with retinopathy. Clinical assessment was performed during levodopa therapy (*on* state) and after treatment discontinuation (*off* state), using the Fahn–Tolosa–Marin Tremor Rating Scale (FTM-TRS) and the MDS-UPDRS Part III. In the untreated state, the clinical assessment showed slight worsening in Archimedes spirals drawing and toe-tapping performance, which accounted for the minimal changes in clinical scores (FTM-TRS: 33 vs. 34; MDS-UPDRS-III: 25 vs. 26), consistent with a subtherapeutic dose.

**Supplementary appendix 2. DAT-SPECT Semi-Quantitative Analysis (DaTQUANT)**

The semi-quantitative analysis of DAT-SPECT imaging was performed using DaTQUANT software (GE Healthcare), which enables standardized and automated quantification of striatal dopamine transporter binding. The software automatically positions predefined voxels of interest (VOIs) over the bilateral caudate and putamen to calculate asymmetry indices and putamen-to-caudate uptake ratios [1]. These uptake values are quantified volumetrically, allowing for comparison across distinct striatal subregions, based on alignment with a standardized anatomical template registered to the patient’s individual SPECT images. Tracer uptake within each VOI was normalized to a reference region located in the occipital cortex—an area with minimal dopamine transporter expression. The specific binding ratio (SBR) for each VOI was computed as follows:
(mean counts in the striatal VOI – mean counts in the background region) / mean counts in the background region. SBR values were obtained for the right and left striatum, caudate, and putamen. In addition, bilateral putamen-to-caudate ratios were calculated. All uptake values were compared against an internal normative database provided by the software and matched for patient age [2]. Diagnostic classification was based on reference values aligned with those proposed by Lanfranchi et al. [3].

**REFERENCES**

[1] J. E. Brogley, «DaTQUANT: The Future of Diagnosing Parkinson Disease», *J. Nucl. Med. Technol.*, vol. 47, fasc. 1, pp. 21–26, mar. 2019, doi: 10.2967/jnmt.118.222349.

[2] D. Colella *et al.*, «Subtle changes in central dopaminergic tone underlie bradykinesia in essential tremor», *NeuroImage Clin.*, vol. 40, p. 103526, 2023, doi: 10.1016/j.nicl.2023.103526.

[3] F. Lanfranchi *et al.*, «Different z-score cut-offs for striatal binding ratio (SBR) of DaT SPECT are needed to support the diagnosis of Parkinson’s Disease (PD) and dementia with Lewy bodies (DLB)», *Eur. J. Nucl. Med. Mol. Imaging*, vol. 50, fasc. 4, pp. 1090–1102, mar. 2023, doi: 10.1007/s00259-022-06069-0.
